# Effect of vitamin D_3_ supplementation on iron status: a randomized, double-blind, placebo-controlled trial among ethnic minorities living in Norway

**DOI:** 10.1186/s12937-016-0192-7

**Published:** 2016-08-09

**Authors:** Ahmed A. Madar, Lars C. Stene, Haakon E. Meyer, Mette Brekke, Per Lagerløv, Kirsten V. Knutsen

**Affiliations:** 1 Department of Community Medicine, Institute of Health and Society, University of Oslo, Oslo, Norway; 2 Department of General Practice, Institute of Health and Society, University of Oslo, Oslo, Norway; 3 Division of Epidemiology, Norwegian Institute of Public Health, Oslo, Norway

**Keywords:** Iron status, Vitamin D, Immigrants, Randomised controlled trials

## Abstract

**Background:**

Both vitamin D and iron deficiencies are widespread globally, and a relationship between these deficiencies has been suggested. However, there is a paucity of randomised controlled trials assessing the effect of vitamin D supplementation on iron status.

**Purpose:**

We aimed to investigate whether 16 weeks of daily vitamin D_3_ supplementation had an effect on serum ferritin, haemoglobin, serum iron and transferrin saturation.

**Methods:**

Overall, 251 participants from South Asia, Middle East and Africa aged 18–50 years who were living in Norway were randomised to receive daily oral supplementation of 10 μg vitamin D_3_, 25 μg vitamin D_3_, or placebo for 16 weeks during the late winter. Blood samples from baseline and after 16 weeks were analysed for serum 25-hydroxyvitamin D (s-25(OH) D), serum ferritin, haemoglobin and serum iron. In total, 214 eligible participants completed the intervention (86 % of those randomised). Linear regression analysis were used to test the effect of vitamin D3 supplementation combined (10 or 25 μg) and separate doses 10 or 25 μg compared to placebo on change (T2-T1) in each outcome variable adjusted for baseline s-25(OH)D values.

**Results:**

There was no difference in change in the levels of s-ferritin (1.9 μg/L, 95 % CI: -3.2, 7.0), haemoglobin (-0.02 g/dL, 95 % CI: -0.12, 0.09), s-iron (0.4 μg/L, 95 % CI: -0.5, 1.3) or transferrin saturation (0.7 %, 95 % CI: -0.6.1, 2.0) between those receiving vitamin D_3_ or those receiving placebo. Serum 25-hydroxyvitamin D increased from 29 nmol/L at baseline to 49 nmol/L after the intervention, with little change in the placebo group.

**Conclusions:**

In this population of healthy ethnic minorities from South Asia, the Middle East and Africa who had low vitamin D status, 16 weeks of daily supplementation with 10 or 25 μg of vitamin D_3_ did not significantly affect the haemoglobin levels or other markers of iron status.

**Electronic supplementary material:**

The online version of this article (doi:10.1186/s12937-016-0192-7) contains supplementary material, which is available to authorized users.

## Introduction

Iron deficiency remains one of the most severe and important nutritional deficiencies in the world. This deficiency affects more than 30 % of the world’s population, thus impacting an estimated 2 billion people [1, 2]. Iron deficiency causes anaemia and disrupts the optimal function of both the endocrine and immune systems. Iron homeostasis is strictly controlled by duodenal enterocytes, which absorb dietary iron; macrophages, which recycle iron from erythrocytes and other cells and hepatocytes, which store iron and can release it when needed [3]. In addition, vitamin D deficiency is widespread, and a high prevalence of vitamin D insufficiency among non-western immigrant populations living in Western countries has been reported [4–8].

A co-existence of vitamin D and iron deficiencies has been reported, and an association between low serum 25-hydroxyvitamin D (s- 25(OH) D) and low levels of serum iron (s-iron), low erythrocyte values and transferrin saturation has been found in cross-sectional studies [9–12]. One study found that 92 % of iron-deficient Spanish women aged 18–35 years also had vitamin D deficiency or insufficiency (defined as an s- 25(OH)D concentration of <50 nmol/L or 51–74 nmol/L respectively) [13]. In addition, an inverse association between iron status and bone resorption in young menstruating women has been reported [14, 15].

The possible mechanism of these associations could involve erythrocyte precursor cells that express 1,25-hydroxyvitamin D (the active form of vitamin D) receptors, which induce the proliferation and maturation of erythroid progenitor cells. Therefore, deficiency of 1,25-hydroxyvitamin D may affect erythropoiesis [16–18].

It has also been documented that hepcidin regulates the absorption, tissue distribution, and extracellular concentration of iron by suppressing the ferroportin-mediated export of cellular iron [19, 20]. Vitamin D is a potent regulator of the hepcidin-ferroportin axis therefore, vitamin D deficiency may affect the regulation of hepcidin, which could accelerate the decrease in haemoglobin and increase the incidence of anaemia [21, 22].

In Norway, vitamin D deficiency is far more prevalent among immigrants than among ethnic Norwegians, and more than one-third of immigrants from the Middle East, Sub-Saharan Africa and South Asia have 25(OH)D below 25 nmol/L [4, 5, 23, 24]. Currently, aside from the results of small studies conducted among Pakistani pregnant women and children in the 1990s, which found a high prevalence of iron deficiency, there are no data on iron status among immigrant populations in Norway [25–27].

Although observational studies have suggested a relationship between these deficiencies, there is a paucity of randomized controlled trials (RCTs) assessing the effect of vitamin D supplementation on iron status. However, in a small randomised, placebo-controlled trial, Indians aged 15 to 60 years with concurrent iron-deficiency anaemia and vitamin D deficiency anaemia received vitamin D_3_ intramuscularly, but the vitamin D supplement did not improve haemoglobin concentrations [28]. The trial was small and non-conclusive, and other studies were cross-sectional.

In addition, randomised studies that examined the effect of vitamin D on iron status in the general immigrant population have not been conducted. We therefore present here results for the predefined additional objective of our previously reported randomised, double-blind, placebo-controlled trial on the effect of vitamin D supplementation on muscle strength and power among ethnic minorities in Norway [29]. We hypothesised that improving the low vitamin D status in immigrants would positively affect their iron status. The aim of the present study was to determine whether 16 weeks of daily vitamin D_3_ supplementation (10 or 25 μg/d) *vs* placebo would affect the iron status (serum ferritin, haemoglobin, s-iron, and transferrin saturation) in a multi-ethnic immigrant population during the late wintertime.

## Materials and methods

The study subjects were healthy men and women aged 18–50 years who were born or whose parents were born in the Middle East, Africa or South Asia. The subjects were recruited from 11 different community centres in Oslo and the surrounding areas (at latitude 60°N). The inclusion criteria included not pregnant, non-breastfeeding, not regularly using vitamin D-containing supplements, not being treated for vitamin D deficiency, not using medication that interfered with vitamin D metabolism (thiazides, anti-epileptics, prednisolone or hormone replacement therapy), and not suffering from any condition such as malabsorption, kidney diseases, cancer, tuberculosis, sarcoidosis, osteoporosis or recent fractures. All of the female participants were younger than 50 years old (when menopause normally starts), but they were not asked about menopausal status or the current use of oral contraception. The same data collection team visited all of the centres and performed the baseline and follow-up data collection. Interpreters were used when necessary, but the majority of the study participants were able to communicate in the Norwegian language.

### Randomisation and intervention

The 16-week intervention study was carried out from January to June 2011. Those who fulfilled the eligibility criteria were randomly assigned to one of three equally sized intervention groups receiving one tablet per day containing 25 μg vitamin D_3_, 10 μg vitamin D_3_ or placebo. The tablets were similar in colour, size and packing. Each participant was given a box containing 120 tablets (a 16-week use corresponds to 112 tablets) at baseline along with a self-administered compliance form. The tablets were manufactured by Bio Plus Life Sciences PVT LTD, DMA (Bangalore, India), which is certified for Good Manufacturing Practice, and the ingredients met the requirements of British Pharmacopé. If the study subjects forgot to take a tablet on one day, they were asked to take two tablets on the following day. Participants were followed up with a short text message twice a week to remind them to take the tablets. Subjects were advised to maintain their usual dietary pattern during the 16-week trial period and were advised to contact the study staff by telephone if they had any queries.

### Main outcome variables

The study outcomes included the changes in the following status during the 16-week intervention between the pooled intervention groups (10 or 25 μg of vitamin D_3_/d) and the placebo group: s-ferritin, haemoglobin, s-iron and transferrin saturation.

### Random allocation

We chose a computer-generated block randomisation method to ensure a good balance between the number of individuals in each group during the trial and randomly varied the block size between 3 and 6.

### Blinding

Group allocation was unknown to participants, research staff, investigators, and data collectors. The tablet boxes were numbered according to the randomisation list by an external pharmacy (the Hospital Pharmacy at Oslo University Hospital). The group allocation list was stored at this pharmacy with a copy of the list in a sealed envelope. Each participant was consecutively numbered and received a pre-packaged tablet box with the corresponding number.

### Registration and ethics approval

The study was authorised as a clinical trial by the Norwegian Medicine Agency and was approved by the Regional Committee for Medical and Health Research Ethics (study code: 2010/1982). All participants gave written informed consent. The study has been registered at EudraCT (2010-021114-36). The clinical trial was conducted according to the principles of the Declaration of Helsinki and in accordance with national laws. Clinical Trials.gov identifier NCT01263288.

### Blood sampling and analyses

Non-fasting venous blood was drawn at baseline and after 16 weeks. To obtain serum, blood was collected in serum-separator gel tubes and was centrifuged after 30 min to 2 h. To collect plasma, blood was collected in EDTA-tubes and centrifuged within 30 min at room temperature at the study site. Serum and plasma were separated and frozen in several aliquots at -20 °C on the same day as sample collection and were transferred at intervals of 1–2 weeks to -80 °C storage until the samples were analysed. After the completion of the study, all serum samples from baseline and follow-up were analysed in one batch at Fürst Medical Laboratory (www.furst.no), which is accredited by the International Organization for Standardisation and is part of the vitamin D quality assessment scheme (DEQAS). Serum 25-hydroxyvitamin D (25(OH) D) was measured using high-pressure liquid chromatography tandem mass spectrometry (HPLC-MS-MS) with the Waters Acquity UPLC and Waters triple quadrupole MS instruments. In house standards at four levels ranging from 25–200 nmol/L were calibrated against external MS-standards from Recipe (Germany), product no. MS7013, traceable to National Institute of Standards and Technology (NIST). A deuterised internal standard with C26,27 hexadeuterium-labelled 25(OH) D_3_, which was purchased from Synthetica (Norway), was used to calculate both 25(OH) D_2_ and 25(OH) D_3_. The CV (reproducibility within the laboratory, 4 instruments) for serum 25(OH) D_3_ was 8 % at a concentration of 55.2 nmol/L and 6 % at a concentration of 195.1 nmol/L. In the analysis, the term 25(OH)D is used for the sum of 25(OH) D_2_ and –D_3_, but we note that the contribution of 25(OH)D_2_ was negligible.

Haemoglobin was measured using SYSMEX, which utilises the non-cyanide reagent, sodium lauryl sulfate (SLS). S-ferritin was analysed using an immunoturbidimetric method, and the total iron binding capacity (TIBC) was calculated (TIBC (μmol/L) = 25.1 × Transferrin (g/L). S-iron was measured by colorimetric assays using Ferrozine. Transferrin saturation (%) was calculated as (iron/TIBC) x 100. Serum folic acid and serum vitamin B_12_ levels were measured using the Centaur XP system from Siemens. The interassay coefficients of variation were 1.7 % (haemoglobin), 2.3 % (ferritin), 2.0 % (TIBC), 2.7 % (iron), 8.2 % (folic acid) and 7.4 % (vitamin B_12_). C-reactive protein (CRP), which is a marker of inflammation in the body, was measured (reference < 5 mg/L).

Anaemia was defined as a haemoglobin concentration of < 12 g/dL for women and <13 g/dL for men. Iron deficiency anaemia (IDA) was defined as a haemoglobin level of <12 g/dL and s-ferritin of <15 μg/L for women and a haemoglobin level of <13 g/dL and s-ferritin of <15 μg/L for men [30, 31]. The s-ferritin level is the most specific biochemical test that correlates with total body iron stores and has been used as a key parameter in several epidemiological studies to assess iron status [32]. Transferrin saturation (TS) is an indication of the ability to bind iron and transport it to various sites such as the bone marrow or liver and serum iron and TIBC are needed to calculate the TS %. We want to include TS and TIBC to capture any suspect of iron disorders (deficiency or overload). Vitamin B12 deficiency frequently causes macrocytic anemia and folate deficiency is characteristically associated with macrocytosis and megaloblastic anaemia. We have recently reported that vitamin D deficiency and secondary hyperparathyroidism (SHPT) is prevalent among non-western immigrants in Norway, it also documented that marrow fibrosis has been reported in cases of primary and secondary hyperparathyroidism with very high levels of PTH.

### Statistical analyses

The sample size was planned for an effect of the intervention on muscle strength and power. The results from the primary endpoint have previously been reported [29] and here we present results from predefined additional endpoints. The sample calculation suggested that we include 210 participants; thus, under the assumption of an expected dropout rate of 15–20 %, we aimed to recruit at least 250 participants. Statistical analysis of the data was performed using the IBM SPSS statistical software (V.19.0; SPSS Inc, Chicago, Illinois, USA). For each of the outcome variables, we calculated the difference in change from baseline to follow-up between the combined/pooled intervention groups (10 or 25 μg/day) and the placebo group. These findings were analysed using linear regression analysis, where the effect on each outcome variable was adjusted for the respective baseline concentration. The same analysis as described above was performed to compare 25 μg/day to placebo and 10 μg/day to placebo separately. *P* values of <0.05 were considered statistically significant. Subgroup analyses by baseline values of end point measures, gender, and intervention dose were also performed. The effect estimates for each outcome variable are the change from baseline to 16 weeks.

## Results

**Fig. 1:**
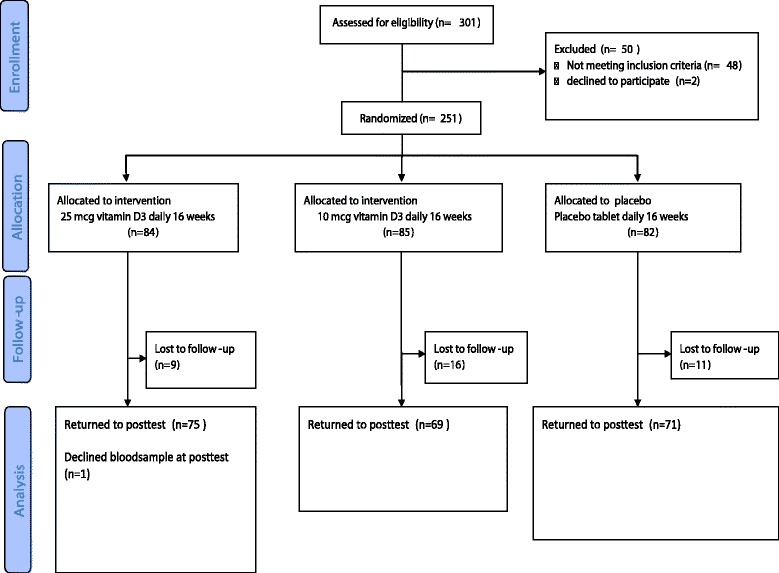
Flow chart of recruitment, randomization and follow-up

301 persons were assessed for eligibility of the study and 251 persons fulfilled the inclusion criteria the participation rate was 83 % and did not differ by ethnicity. A total of 251 study participants were randomly assigned to one of the three interventions. After 16 weeks, 214 (85 %) study participants completed the study (Fig. 1). Participant characteristics are shown in Table 1. No substantial between-group differences in baseline values were noted (Table 1). The baseline characteristics of the 37 participants who did not complete the study were not different from those who completed the study (Additional file 1: Table S1). Although the study participants originated from 11 different countries in the Middle East, Sub- Sahara Africa and South Asia, persons with Somali (*n* = 97) and Tamil background were the majority (*n* = 71).

**Table 1:** Baseline characteristic for the 251 participants who completed the baseline tests^a^

	25 μg Vitamin D3	10 μg Vitamin D3	Placebo
	*N* = 84	*N* = 85	*N* = 82
Age (years)	36.4 (8.2)	37.0 (7.6)	38.5 (7.6)
Gender, female N (%)	58 (69)	61 (72)	63 (77)
Region of origin N (%)			
South Asia	31 (37)	31 (36)	33 (40)
Middle East and North Africa	15 (18)	9 (11)	12 (15)
Sub-Sahara Africa	38 (45)	45 (53)	37 (45)
Body mass index	27.0 (5.2)	27.5(5.2)	27.8 (5.0)
Female	27.7 (5.3)	27.7 (5.3)	28.9 (5.0)
Male	25.0 (4.6)	26.3 (3.0)	24.5 (3,3)
S- 25(OH)D (nmol/L)	26.9 (16.4)	29.7 (20.5)	30.0 (19.0)
Female	26.6 (17.3)	30.8 (22.3)	30.6 (19.8)
Male	27.5 (14.6)	26.9 (15.2)	28.6 (16.2)
Hemoglobin (g/dL)^b^	13.6 (1.6)	13.7 (1.6)	13.6 (1.4)
Female	12.8 (1.2)	13.1 (1.4)	13.0 (0.9)
Male	15.2 (0.9)	15.1 (1.2)	15.5 (0.8)
Serum iron (μg/L)^c^	14.4 (6.6)	14.3 (5.7)	13.1 (5.7)
Female	12.5 (5.5)	13.6 (5.8)	12.0 (5.4)
Male	18.6 (6.8)	15.8 (5.3)	16.9 (4.9)
Serum ferritin (μg/L)^c^ mean (SD)	58.9 (62.8)	81.6 (88.8)	56.3 (63.2)
Female	33.1 (38.8)	46.4 (40.5)	34.1 (41.6)
Male	116.7 (68.2)	171.4 (113.3)	129.6 (68.2)
Serum ferritin (μg/L) median (IQR)			
Female	22 (9.5–46.8)	36 (21–59)	24 (10–34.0)
Male	104 (61–168)	145 (102–219)	110 (82–153)
Transferrin saturation (%)^d^	20.8 (10.4)	21.5 (9.3)	18.4 (8.4)
Female	17.6 (8.8))	20.7 (10.0)	16.5 (7.8)
Male	27.7 (10.3)	23.4 (7.4)	24.9 (6.4)
S-Total iron binding bapacity (μmol/L)^c^	72.1 (11.8)	68.6 (10.9)	72.9 (10.8)
Female	74.0 (12.3)	68.9 (11.3)	74.3 (10.9)
Male	67.6 (9.3)	67.5 (9.9)	68.3 (9.4)
S-Folic acid (nmol/L)^c^	14.8 (6.2)	15.9 (6.6)	15.8 (5.8)
Female	15.5 (6.6)	15.3 (6.0)	15.9 (6.0)
Male	13.4 (5.3)	17.8 (7.9)	15.6 (4.7)
S-Vitamin B12 (pmol/L)^c^	321.6 (92.7)	343.9 (116.8)	339.9 (105.5)
Female	334.1 (93.8)	359.2 (119.8)	334.1 (110.9)
Male	293.8 (85.9)	300.7 (98.6)	361.4 (82.1)
Plasma PTH (pmol/L)			
Female	7.3 (3.1)	7.5 (3.8)	8.5 (4.3)
Male	8.0 (3.6)	6.8 (2.4)	6.3 (2.6)
S-CRP (mg/L)^e^			
All	4.9 (6.5)	4.0 (5.5)	4.2 (7.7)
All (median, IQR)	1.8 (0.8–6.7)	1.8 (0.9–4.0)	2.0 (0.8–4.0)

^a^Data are mean (SD) unless specified otherwise

^b^ *N* = 209

^c^Reference range; s-ferritin (women and men > 16 years 15 -200 and 20 - 300 μg/L respectively), hemoglobin (women and men > 14 years 12–15.3 and 13.4–17), s-iron (women and men > 18 years 9–34 μmol/L), Transferrin saturation (women < 50 years 10–50 % and men 15–57 %), s-Folic acid >5.7 nmol/L and for s-vitamin B12 (170–650 pmol/L)

^d^ Iron/TIBC x 100

^e^ Serum C-reactive protein (CRP) included high-sensitivity CRP (hs CRP)

### Baseline characteristics

The mean baseline s-25(OH)D concentration for the whole study population was 29 (SD 17.6) nmol/L. Around 90 and 53 % of the participants had an s-25(OH)D of <50 and s-25(OH)D of <25 nmol/L respectively. The mean baseline haemoglobin concentration for the study population was 13.7 (SD 1.5). In the sample, approximately 14 % (*n* = 21) of females and 5 % (*n* = 3) of males had anaemia, while 29 % (*n* = 45) of females and 4 % (*n* = 2) of males had low serum ferritin values (<15 μg/L) values. Furthermore 10 % (*n* = 16) of females and none of the males had iron deficiency anaemia. There were no significant correlations between the baseline levels of s-25(OH)D and s-ferritin (*r* = 0.06), haemoglobin (*r* = 0.04), transferrin saturation (*r* = -0.004) or s-iron (*r* = -0.05). CRP values were generally low, but approximately 23 % of the subjects had a CRP > 5 mg/L. All of the participants had normal s-vitamin B12 and s-folic acid levels (Table 1).

### Effect of vitamin D supplementation on endpoint measures

Vitamin D_3_ supplementation (10 or 25 μg combined compared to placebo) for 16 weeks had no statistically significant effects on s-ferritin, haemoglobin, s-iron, or transferrin saturation (Table 2).

**Table 2:** Effect of vitamin D supplementation combined (10 μg or 25 μg)^a^ on serum ferritin, hemoglobin, serum iron, Transferrin Saturation, folic acid and vitamin B12

	Baseline^d^	After 16 weeks	Change from baseline to 16 weeks	Difference (95 % CI) compared to placebo^b^	*P*-value^e^
S-25(OHD) (nmol/l)					
Intervention (*n* = 143)	28.7 (18.6)	48.8 (19.6)	20.1 (21.7)	21.3 (16.7, 26.0)	<0.0001
Placebo (*n* = 71)	29.2 (15.6)	27.5 (13.7)	-1.5 (11.3)		
Hemoglobin (g/dL)^c^					
Intervention (*n* = 136)	13.7 (1.5)	13.5 (1.6)	-0.21 (0.6)	-0.02 (-0.12, 0.09)	NS
Placebo (*n* = 69)	13.5 (1.4)	13.3 (1.4)	-0.27 (0.7)		
Serum ferritin (μg/L)					
Intervention (*n* = 143)	68.4 (77.3)	68.0 (85.5)	-0.3 (35.7)	1.9 (-3.2, 7.0)	NS
Placebo (*n* = 71)	54.4 (63.2)	51.0 (57.8)	-3.4 (19.1)		
Serum iron (μg/L)					
Intervention (*n* = 143)	14.4 (5.8)	14.1 (6.1)	-0.4 (5.9)	0.4 (-0.5, 1.3)	NS
Placebo (*n* = 71)	13.0 (5.9)	13.5 (6.7)	0.5 (5.9)		
Transferrin saturation (%)					
Intervention (*n* = 143)	21.3 (9.4)	20.6 (9.7)	0.5 (8.5)	0.7 (-0.6, 2.0)	NS
Placebo (*n* = 71)	18.3 (8.7)	18.7 (9.5)	-0.7 (8.6)		
S-Total iron binding capacity (nmol/L)					
Intervention (*n* = 143)	70.3 (11.5)	70.8 (11.5)	0.5 (5.5)	-0.08 (-1.0, 0.8)	NS
Placebo (*n* = 71)	73.2 (10.8)	73.5 (11.1)	0.4 (6.1)		
S-Folic acid (nmol/L)					
Intervention (*n* = 143)	15.6 (6.9)	15.0 (7.2)	-0.5 (5.1)	-0.1 (-1.0, 0.7)	NS
Placebo (*n* = 71)	15.8 (5.7)	15.7 (6.0)	-0.1 (6.1)		
S-Vitamin B12 (pmol/L)					
Intervention (*n* = 143)	334.9 (105))	323.3 (112.9)	-9.1 (64.9)	-5.4 (-16.1, 5.3)	NS
Placebo (*n* = 71)	339.9 (105)	337.2 (118.9)	-2.7 (67.7)		

^a^The two intervention groups (10 μg or 25 μg) were combined and compared to the placebo group

^b^Linear regression was used to compare the changes of outcome variables between intervention and placebo adjusted for baseline level of s-25(OH)D and to calculate the *p*-value

^c^ *N* = 205

^d^Data are mean (standard deviation) unless specified otherwise

^e^Apart from s-25(OH) D change from baseline to study end was not significant for any of the parameters in the table

### Effects of vitamin D supplementation on serum 25(OH) D, folic acid and vitamin B12, CRP

As previously reported [29], following the intervention, the mean s-25(OH)D levels increased by a mean of 17 and 26 nmol/L for the 10 μg and 25 μg vitamin D_3_ groups, respectively, compared to placebo (Table 2). There was no change in s-vitamin B12 and s-folic acid levels as a result of vitamin D supplementation (Table 2). As previously reported ([33]) there was no difference in change of s-CRP (mean difference: 0.09 mg/L (95 % CI: − 1.1, 1.4, *P* = 0.9)).

We also analysed the effect of the two interventions (10 and 25 μg) separately, and none of the dosages had a significant effect on the iron status (Table 3). Vitamin D_3_ supplementation had also no effect on s-folic acid and s-vitamin B12 levels.

**Table 3:** Effect of intervention with 10 and 25 μg vitamin D3 supplementation on serum ferritin, hemoglobin, serum iron, Transferrin saturation, folic acid and vitamin B12

	Baseline^b^			Final (after 16 weeks)^b^			Difference in change (95 % CI)^a^	
	Placebo	10 μg	25 μg	Placebo	10 μg	25 μg	10 μg	25 μg
S-25(OHD) (nmol/L)	29.2 (15.6)	30.7 (20.1)	26.8 (17.1)	27.5 (13.7)	45.9 (18.7)	51.1 (20.1)	17.5 (12.8, 22.1)	25.0 (20.4, 31.9)^*^
Hemoglobin (g/dL)	13.5 (1.4)	13.8 (1.7)	13.7 (1.4)	13.3 (1.4)	13.5 (1.6)	13.5 (1.6)	-0.07 (-0.27, 0.14)	-0.06 (-0.25, 0.1)
Serum ferritin (μg/L)	54.4 (63.2)	82.6 (92.5)	55.1 (57.2)	51.0 (57.8)	81.5 (106.2)	55.5 (58.0)	2.98 (-9.3, 15.3)	3.7 (-2.6, 9.9)
Serum iron (μg/L)	13.0 (5.9)	14.4 (5.9)	14.5 (5.8)	13.5 (6.7)	13.0 (5.5)	15.1 (6.4)	-1.3.(-3.0, 0.5)	-0.6 (-2.3, 1.0)
Transferrin saturation (%)	18.3 (.8.7)	21.7 (9.5)	20.9 (9.3)	18.7 (9.6)	19.3 (8.9)	21.7 (10.2)	-1.3 (-3.9, 1.3)	-1.0 (-3.4, 1.4)
S-Total iron binding capacity (nmol/L)	73.2 (10.8)	68.7 (11.1)	71.7 (11.7)	73.5 (11.1)	69.4 (11.1)	72.1 (12.0)	-0.23 (-2.2, 1.7)	0.2 (-1.6, 2.0)
S-Folic acid (nmol/L)	15.8 (5.8)	15.9 (6.6)	15.2 (7.1)	15.7 (6.1)	14.9 (7.1)	15.1 (7.3)	-0.9 (-2.5, 0.7)	-0.14 (-1.1, 0.8)
S-Vitamin B12 (pmol/L)	339.9 (105.5)	343.9 (116.8)	321.6 (92.7)	337.2 (118.9)	337.6 (132.7)	310 (89.5)	-3.3 (-28.1, 21.5)	-5.5 (-14.8, 3.9)

* *p* < 0.0001

^a^difference in change comparing to placebo and adjusted for baseline s-25(OH)D values

^b^Data are mean (standard deviation) unless specified otherwise

### Additional analyses

We have conducted analysis in the sub-group with anaemia and vitamin D3 supplementation had no effect on iron status. Additional analyses resulted in similar findings that were independent of whether the baseline concentration of the iron status was above or below the mean among participants. Also, analyses ignoring the randomized design, we found no significant associations between changes in s-25(OH)D and changes in serum indicators from baseline to 16 weeks in the total study population (data not shown). The results were also consistent after stratification of the data by gender, region of origin or a parathyroid hormone (PTH) greater than the reference value (data not shown).

### Compliance

Compliance with supplementation was confirmed by counting the number of tablets in the returned tablet boxes, where 80 % had consumed more than 80 % of the tablets and 69 % had consumed more than 90 % of the tablets.

## Discussion

The present study showed that 16 weeks of daily vitamin D_3_ supplementation (10 or 25 μg) to healthy ethnic minorities did not significantly affect serum ferritin, haemoglobin, s-iron or transferrin saturation. To our knowledge, this is the first randomised controlled trial studying the effect of vitamin D supplementation on iron status in a presumed healthy immigrant population. We found that at baseline, 14 % of females and 5 % of males had anaemia, whereas a third of females and 4 % of males had depleted iron stores. Furthermore, 90 % of the study subjects had a 25(OH)D concentration of <50 nmol/L. At baseline the mean s-25(OH)D among the 21 females with haemoglobin below 11 g/dL was 23.7 nmol/L which is not so much different for the levels of s-25(OH)D in the whole study population. However, the vitamin D3 supplementation for 16 weeks had no statistically significant effects on haemoglobin or any of other endpoints in this small group with iron deficiency.

New insights into the biologic functions of vitamin D have led to increased interest in the clinical consequences of vitamin D deficiency. In addition, a number of cross-sectional studies have demonstrated an association between low 25-hydroxyvitamin D levels and poor iron status [9, 11, 13, 34]. To evaluate the prevalence of anaemia in a population of individuals with vitamin D deficiency, Sim et al. studied 554 subjects in a general population over a period of 2 years as part of normal healthcare operations. Their study demonstrates an association between vitamin D deficiency, a greater risk of anaemia, lower mean haemoglobin levels, and a higher use of the erythropoiesis-stimulating agent [9]. These findings suggested that vitamin D can have an effect on erythropoiesis, where the 1,25 hydroxyvitamin D hormone plays a role in the proliferation and maturation of erythroid progenitor cells, therefore, a deficiency of 1,25 hydroxyvitamin D hormone may impair erythropoiesis [17, 18].

Few studies have evaluated the effect of vitamin D supplementation on iron status in immigrant populations. Our findings are in agreement with the results from a small randomised controlled study in Indians (*n* = 30, 15–60 years) with iron-deficiency anaemia who received one dose of vitamin D_3_ (0.6 million units intramuscularly) and parental iron [28]. Another randomised placebo-controlled study involved menstruating Spanish women (*n* = 109, 18–35 years) who were given iron or iron along with vitamin D to study the effect on iron metabolism. The results showed higher values of haematological parameters in the iron plus vitamin D group compared to the iron only group, indicating that vitamin D could enhance iron status when taken together with iron [35].

Furthermore, the role of vitamin D in erythropoiesis has been suggested by clinical observations. Supplementation with ergocalciferol has been associated with dose reductions in erythropoiesis-stimulating agents and increased reticulocytosis in haemodialysis patients [36]. Prolonged infection or inflammation often leads to the development of anemia (anemia of inflammation) and there is a growing literature evaluating the effect of vitamin D3 on inflammation [37]. However, our results support the findings from a recent and large randomised placebo-controlled trial that examined the impact of a 3-month period of oral vitamin D supplementation on circulating inflammatory markers in an African-American cohort where vitamin D_3_ supplementation did not affect CRP levels. [38]. Also, a meta-analysis of RCTs in otherwise healthy individuals showed no effect of vitamin D supplementation on CRP or other markers of inflammation [39].

Furthermore, in patients with chronic kidney disease, low levels of s-25(OH)D and 1,25-hydroxyvitamin D (1,25(OH)D), the active form of vitamin D, were found to be independently associated with decreased haemoglobin levels and anaemia [17].

Additionally, marrow fibrosis has been reported in cases of primary and secondary hyperparathyroidism with very high levels of PTH. It has been suggested that vitamin D improves anaemia only in those with very high PTH levels and those with marrow fibrosis [40]. However, in our study, we did not find any correlation between baseline s-ferritin or haemoglobin concentration and the levels of PTH. Observational studies show associations that are indirectly due to poor diet is low in both bioavailable iron and vitamin D. There are few possible reasons for the lack of effect of vitamin D supplementation on iron status in our study. Is it possible that this study did not find any effects of vitamin D supplementation on iron status because the bulk of the participants were relatively sufficient in iron and only a small proportion of the participants had anaemia (*n* = 21). However, our sub-group analysis among the proportion with anaemia the vitamin D3 supplementation had no effect on iron status. Another possible explanation is that we don’t have sufficient power to detect any changes because the sample size was planned for an effect of the intervention on muscle strength and power. It is also proposed that it is likely that vitamin D improves anaemia only in those with very high PTH and marrow fibrosis. In our study over 30 % had PTH levels above the upper limit of the reference value at baseline, but effect of vitamin D supplementation was not different than those with normal PTH.

### Strengths and weaknesses

The strengths of this study included the following: it was a strictly performed double-blind randomised placebo-controlled trial, had good compliance and had relatively high retention. Assessments were performed during the winter and spring, at a time when sun exposure has little impact on vitamin D synthesis, and all blood samples were assayed in a single batch. The ethnic minorities targeted in our study are known to have generally poor vitamin D status. We measured the main iron status to assess anaemia and iron deficiency anaemia. The study also had some limitations. Although the vitamin D doses used were sufficient to increase the s-25(OH)D levels, the recommended level of 50 nmol/l was not reached in 43 % of the 25 μg supplementation group or in 62 % of the 10 μg supplementation group. In addition, although the analyses of iron markers were pre-planned, the study was designed primarily to examine the outcomes on muscular strength. We have not collected information about the whole diet and therefore could not assess the dietary intake of iron, vitamin B12 and folate.

Iron deficiency anaemia is the last end stage of progression from normal iron status to frank deficiency. Iron deficiency reduces the work capacity of individuals and has adverse effects on the immune system (high risk of infection) and several other chronic conditions [41–43]. Women of reproductive age are at particularly high risk of iron deficiency and its consequences. Therefore, a package of public health measures addressing all aspects of iron deficiency, anaemia and vitamin D deficiency are needed. The study can be generalized to other immigrant populations in Europe, but not necessarily to all ethnic groups or populations with anaemia and more research in populations with anaemia is needed.

## Conclusion

The main finding of this study was that supplementation with vitamin D_3_ over 16 weeks did not improve serum ferritin, haemoglobin, s-iron or transferrin saturation in healthy adults. This study suggests that vitamin D supplementation does not improve iron status for people without anaemia.

## Abbreviations

CRP, C-reactive protein; DEQAS, International Organization for Standardisation and is part of the vitamin D quality assessment scheme; HPLC-MS-MS, High-pressure Liquid Chromatography tandem Mass Spectrometry; IDA, Iron Deficiency Anaemia TIBC: Iron Binding Capacity; PTH, Parathyroid Hormone; RCTs, Randomised Controlled Trials; S- 25(OH) D, serum 25-hydroxyvitamin D; TS, Transferrin saturation.

## Additional file

Additional file 1: Table S1. Baseline characteristics of the study subjects who did not complete the study. (DOCX 15 kb)

## References

[1] McLean E, Cogswell M, Egli I, Wojdyla D, de Benoist B (2009). Worldwide prevalence of anaemia, WHO Vitamin and Mineral Nutrition Information System, 1993–2005. Public Health Nutr.

[2] Camaschella C (2015). Iron-deficiency anemia. N Engl J Med.

[3] Ganz T (2013). Systemic iron homeostasis. Physiol Rev.

[4] Madar AA, Stene LC, Meyer HE (2009). Vitamin D status among immigrant mothers from Pakistan, Turkey and Somalia and their infants attending child health clinics in Norway. Br J Nutr.

[5] Eggemoen AR, Knutsen KV, Dalen I, Jenum AK (2013). Vitamin D status in recently arrived immigrants from Africa and Asia: a cross-sectional study from Norway of children, adolescents and adults. BMJ Open.

[6] Andersen R, Molgaard C, Skovgaard LT, Brot C, Cashman KD, Jakobsen J, Lamberg-Allardt C, Ovesen L (2008). Pakistani immigrant children and adults in Denmark have severely low vitamin D status. Eur J Clin Nutr.

[7] Bergstrom I, Palmer M, Persson J, Blanck A (2014). Observational study of vitamin D levels and pain in pregnant immigrant women living in Sweden. Gynecol Endocrinol.

[8] Lips P (2007). Vitamin D status and nutrition in Europe and Asia. J Steroid Biochem Mol Biol.

[9] Sim JJ, Lac PT, Liu IL, Meguerditchian SO, Kumar VA, Kujubu DA, Rasgon SA (2010). Vitamin D deficiency and anemia: a cross-sectional study. Ann Hematol.

[10] Grindulis H, Scott PH, Belton NR, Wharton BA (1986). Combined deficiency of iron and vitamin D in Asian toddlers. Arch Dis Child.

[11] McGillivray G, Skull SA, Davie G, Kofoed SE, Frydenberg A, Rice J, Cooke R, Carapetis JR (2007). High prevalence of asymptomatic vitamin D and iron deficiency in East African immigrant children and adolescents living in a temperate climate. Arch Dis Child.

[12] Morrone A, Nosotti L, Piombo L, Scardella P, Spada R, Pitidis A (2012). Iron deficiency anaemia prevalence in a population of immigrated women in Italy. Eur J Public Health.

[13] Blanco-Rojo R, Perez-Granados AM, Toxqui L, Zazo P, de la Piedra C, Vaquero MP (2013). Relationship between vitamin D deficiency, bone remodelling and iron status in iron-deficient young women consuming an iron-fortified food. Eur J Nutr.

[14] Wright I, Blanco-Rojo R, Fernandez MC, Toxqui L, Moreno G, Perez-Granados AM, de la Piedra C, Remacha ÁF, Vaquero MP (2013). Bone remodelling is reduced by recovery from iron-deficiency anaemia in premenopausal women. J Physiol Biochem.

[15] Toxqui L, Perez-Granados AM, Blanco-Rojo R, Wright I, de la Piedra C, Vaquero MP (2014). Low iron status as a factor of increased bone resorption and effects of an iron and vitamin D-fortified skimmed milk on bone remodelling in young Spanish women. Eur J Nutr.

[16] Alon DB, Chaimovitz C, Dvilansky A, Lugassy G, Douvdevani A, Shany S, Nathan I (2002). Novel role of 1,25(OH)(2)D(3) in induction of erythroid progenitor cell proliferation. Exp Hematol.

[17] Santoro D, Caccamo D, Lucisano S, Buemi M, Sebekova K, Teta D, De Nicola L (2015). Interplay of Vitamin D, Erythropoiesis, and the Renin-Angiotensin. System Biomed Res Int.

[18] Lucisano S, Di Mauro E, Montalto G, Cernaro V, Buemi M, Santoro D (2014). Vitamin D and anemia. J Ren Nutr.

[19] Ganz T, Nemeth E (2012). Hepcidin and iron homeostasis. Biochim Biophys Acta.

[20] Zhao N, Zhang AS, Enns CA (2013). Iron regulation by hepcidin. J Clin Invest.

[21] Bacchetta J, Zaritsky JJ, Sea JL, Chun RF, Lisse TS, Zavala K (2014). Suppression of iron-regulatory hepcidin by vitamin D. J Am Soc Nephrol.

[22] Zughaier SM, Alvarez JA, Sloan JH, Konrad RJ, Tangpricha V (2014). The role of vitamin D in regulating the iron-hepcidin-ferroportin axis in monocytes. J Clin Transl Endocrinol.

[23] Holvik K, Meyer HE, Haug E, Brunvand L (2005). Prevalence and predictors of vitamin D deficiency in five immigrant groups living in Oslo, Norway: the Oslo Immigrant Health Study. Eur J Clin Nutr.

[24] Knutsen KV, Brekke M, Gjelstad S, Lagerlov P (2010). Vitamin D status in patients with musculoskeletal pain, fatigue and headache: a cross-sectional descriptive study in a multi-ethnic general practice in Norway. Scand J Prim Health Care.

[25] Brunvand L, Henriksen C, Larsson M, Sandberg AS (1995). Iron deficiency among pregnant Pakistanis in Norway and the content of phytic acid in their diet. Acta Obstet Gynecol Scand.

[26] Wandell PE (2013). Population groups in dietary transition. Food Nutr Res.

[27] Brunvand L, Sander J (1993). Iron deficiency anemia among immigrant children from developing countries. Tidsskr Nor Laegeforen.

[28] Sooragonda B, Bhadada SK, Shah VN, Malhotra P, Ahluwalia J, Sachdeva N (2015). Effect of vitamin D replacement on hemoglobin concentration in subjects with concurrent iron-deficiency anemia and vitamin D deficiency: a randomized, single-blinded, placebo-controlled trial. Acta Haematol.

[29] Knutsen KV, Madar AA, Lagerlov P, Brekke M, Raastad T, Stene LC, Meyer HE (2014). Does vitamin D improve muscle strength in adults? A randomized, double-blind, placebo-controlled trial among ethnic minorities in Norway. J Clin Endocrinol Metab.

[30] WHO (2008). Worldwide prevalence of anaemia 1993-2005. http://www.who.int/vmnis/publications/anaemia_prevalence/en. Accessed 20 June 2016.

[31] Nordic Council of Ministers (2013). Nordic Nutrition Recommendations. http://www.ravitsemusneuvottelukunta.fi/files/images/vrn/9789289326292_nnr-2012.pdf. Accessed 20 June 2016.

[32] Guyatt GH, Oxman AD, Ali M, Willan A, McIlroy W, Patterson C (1992). Laboratory diagnosis of iron-deficiency anemia. J Gen Intern Med.

[33] Knutsen KV, Madar AA, Brekke M, Meyer HE, Natvig B, Mdala I, Lagerløv P (2014). Effect of vitamin D on musculoskeletal pain and headache: A randomized, double-blind, placebo-controlled trial among adult ethnic minorities in Norway. Pain.

[34] Lee JA, Hwang JS, Hwang IT, Kim DH, Seo JH, Lim JS (2015). Low vitamin D levels are associated with both iron deficiency and anemia in children and adolescents. Pediatr Hematol Oncol.

[35] Toxqui L, Perez-Granados AM, Blanco-Rojo R, Wright I, Gonzalez-Vizcayno C, Vaquero MP (2013). Effects of an iron or iron and vitamin D-fortified flavored skim milk on iron metabolism: a randomized controlled double-blind trial in iron-deficient women. J Am Coll Nutr.

[36] Saab G, Young DO, Gincherman Y, Giles K, Norwood K, Coyne DW (2007). Prevalence of vitamin D deficiency and the safety and effectiveness of monthly ergocalciferol in hemodialysis patients. Nephron Clin Pract.

[37] Smith EM, Alvarez JA, Martin GS, Zughaier SM, Ziegler TR, Tangpricha V (2015). Vitamin D deficiency is associated with anaemia among African Americans in a US cohort. Br J Nutr.

[38] Chandler PD, Scott JB, Drake BF, Ng K, Manson JE, Rifai N (2014). Impact of vitamin D supplementation on inflammatory markers in African Americans: results of a four-arm, randomized, placebo-controlled trial. Cancer Prev Res (Phila).

[39] Autier P, Boniol M, Pizot C, Mullie P (2014). Vitamin D status and ill health: a systematic review. Lancet Diabetes Endocrinol.

[40] Bhadada SK, Bhansali A, Ahluwalia J, Chanukya GV, Behera A, Dutta P (2009). Anaemia and marrow fibrosis in patients with primary hyperparathyroidism before and after curative parathyroidectomy. Clin Endocrinol (Oxf).

[41] Stack AG, Mutwali AI, Nguyen HT, Cronin CJ, Casserly LF, Ferguson J (2014). Transferrin saturation ratio and risk of total and cardiovascular mortality in the general population. QJM.

[42] Brutsaert TD, Hernandez-Cordero S, Rivera J, Viola T, Hughes G, Haas JD (2003). Iron supplementation improves progressive fatigue resistance during dynamic knee extensor exercise in iron-depleted, nonanemic women. Am J Clin Nutr.

[43] Brownlie T, Utermohlen V, Hinton PS, Giordano C, Haas JD (2002). Marginal iron deficiency without anemia impairs aerobic adaptation among previously untrained women. Am J Clin Nutr.

