# Supplementary material for: Effect of vitamin D3 supplementation on iron status: a randomized, double-blind, placebo-controlled trial among ethnic minorities living in Norway
Source: Nutr J. 2016 Aug 9;15:74. doi: 10.1186/s12937-016-0192-7 (PMC4977672; doi:10.1186/s12937-016-0192-7)
Supplement: Additional file 1: Table S1. — Baseline characteristics of the study subjects who did not complete the study. (DOCX 15 kb) [file 12937_2016_192_MOESM1_ESM.docx]

Supplementary table 1. Baseline characteristics of the study subjects who did not complete the study

| Characteristics | Placebo  N= 11 | Vitamin D (10 µg)  N= 16 | Vitamin D (25 µg)  N= 10 |
| --- | --- | --- | --- |
| Age, years (SD) | 35 (8.5) | 37 (8.4) | 35 (8.7) |
| Sex  Male (n, %)  Female (n, %) | 4 (36)  7 (64) | 6 (38)  10 (62) | 3 (30)  7 (70) |
| Ethnic origin  South Asia  Middle East & North Africa  Sub-Sahara Africa | 6  0  5 | 6  2  8 | 2  3  5 |
| Time lived in Norway (years) | 12.0 (8.7) | 14.8 (7.9) | 13.6 (7.4) |
| Level of education (n)  ≤ 10 years  11-13 years  ≥ 14 years | 3  8  0 | 7  5  4 | 5  4  1 |
| S-25(OH)D (nmol/l) | 30 (20) | 30 (22) | 27 (16) |
| Hb (g/dL) | 14.0 (1.5) | 13.6 (1.5) | 12.6 (2.4) |
| Serum ferritin (μg/L) | 68.4(65.4) | 77.8(73.6) | 87.5(94.2) |
| Serum iron (μg/L) | 13.6(4) | 13.6 (5) | 13.5 (11) |
| Transferrin saturation | 19.5(5.7) | 20.7 (9.0) | 19.9(17.0) |
| S-Vitamin B12 (pmol/L) | 319.5 (125) | 280 (77.2) | 320(73) |
| S-Folic acid (nmol/L) | 14(7) | 14 (4) | 14(9) |
